# Evaluating Lacrimal Punctum Size as a Clinical Indicator of Dry Eye Disease Severity in a Real-World Lebanese Cohort

**DOI:** 10.3390/jcm15134987

**Published:** 2026-06-26

**Authors:** Yehya Tlaiss, John Warrak, Elias Warrak

**Affiliations:** 1 Department of Ophthalmology, University of Balamand, Beirut 1107 2020, Lebanon; john.warrak@std.balamand.edu.lb; 2 Advanced Eye Care Center, University of Balamand, Beirut 1107 2020, Lebanon

**Keywords:** dry eye disease, lacrimal punctum, punctum size, tear break-up time, silicone plug, punctal occlusion, ocular surface disease, TFOS DEWS II, tear drainage

## Abstract

**Background/Objectives**: To investigate the relationship between lacrimal punctum size and the severity of dry eye disease (DED) in a clinically refractory, real-world patient cohort from a tertiary ophthalmology center in Lebanon. **Methods**: A retrospective observational study was conducted at Advanced Eye Care Center, Beirut, Lebanon (2016–2024). A total of 312 eyes from 156 patients with moderate-to-severe DED unresponsive to topical artificial tears, loteprednol etabonate, and cyclosporine (0.05% ophthalmic emulsion) were included. All eyes subsequently underwent lower lacrimal punctum plug insertion as part of clinical management. Lacrimal punctal diameter was estimated by the largest silicone plug (0.5 mm, 0.6 mm, or 0.7 mm) inserted nonforcefully into the lower punctum under slit-lamp visualization. Tear film stability was assessed by Tear Break-Up Time (TBUT). Group differences were analyzed using the Kruskal–Wallis H test with post hoc Mann–Whitney U tests (Bonferroni correction), and Spearman’s rank correlation was calculated to quantify the monotonic association. **Results**: Eyes were distributed across punctal diameter categories as follows: 0.5 mm (*n* = 15, 4.8%), 0.6 mm (*n* = 204, 65.4%), and 0.7 mm (*n* = 93, 29.8%). Median TBUT values were 5.55 s [IQR 5.51–5.77], 5.06 s [IQR 4.80–5.37], and 4.51 s [IQR 4.23–4.71] for the 0.5 mm, 0.6 mm, and 0.7 mm groups, respectively. Kruskal–Wallis analysis confirmed significant inter-group differences (H = 140.1, *p* < 0.001). All post hoc pairwise comparisons remained significant after Bonferroni correction (*p* < 0.001). Spearman’s rank correlation demonstrated a significant negative association between lacrimal punctal diameter and TBUT (ρ = −0.70, *p* < 0.000001). All analyses were conducted at the eye level and do not account for within-patient correlation (bilateral design, 156 patients); *p*-values should be interpreted accordingly. **Conclusions**: In this treatment-refractory cohort, larger lacrimal punctal diameter was significantly associated with greater tear film instability. These findings suggest that lacrimal punctal diameter estimation during therapeutic plug insertion may serve as a practical, cost-free adjunct to standard DED evaluation. Prospective multimodal studies are needed to validate punctal diameter as an independent clinical indicator of DED severity.

## 1. Introduction

Dry eye disease (DED) is a prevalent, multifactorial condition characterized by disruption of tear film homeostasis, with global prevalence estimates ranging from 5% to over 50% depending on diagnostic criteria, geographic region, and study population [1,2,3,4]. It constitutes one of the most frequent diagnoses in ophthalmic practice, imposing substantial burdens on visual function, ocular comfort, and quality of life [5,6]. The Tear Film and Ocular Surface Society Dry Eye Workshop II (TFOS DEWS II) defines DED as a multifactorial disease of the ocular surface in which loss of tear film homeostasis is accompanied by ocular symptoms and potential ocular surface damage [7,8].

The pathophysiology of DED encompasses two principal mechanisms: aqueous-deficient dry eye (ADDE), driven by lacrimal gland hypofunction, and evaporative dry eye (EDE), predominantly secondary to meibomian gland dysfunction [9,10,11]. Both pathways converge on elevated tear film osmolarity, ocular surface inflammation, and epithelial barrier disruption [10]. Standard first-line management includes topical lubricants, anti-inflammatory agents, and immunomodulators such as cyclosporine [12,13]; however, a significant proportion of patients with moderate-to-severe ADDE fail to achieve adequate symptom control despite these interventions [6,12].

For this refractory population, punctal occlusion—via silicone plug insertion or thermal cautery—represents an established therapeutic strategy for reducing tear drainage and extending tear film residence time on the ocular surface [2,12]. Beyond their therapeutic function, silicone plugs afford a pragmatic, non-invasive means of estimating lacrimal punctum diameter, an anatomical variable that has received limited systematic clinical attention despite its physiological relevance. Larger punctal openings may promote increased tear outflow, potentially accelerating the breakdown of an already-compromised tear film [14,15].

The limited existing literature on punctum size in DED was pioneered by Chen et al. in a 2021 cross-sectional study of 139 eyes, which employed silicone plug sizes ranging from 0.3 to 0.5 mm and identified a statistically significant but modest positive correlation between larger punctum size and DED severity (ρ = 0.16) [15]. That study, however, did not assess puncta beyond 0.5 mm and included a heterogeneous population not restricted to treatment-refractory cases, leaving an important clinical gap.

The present study addresses these limitations by evaluating a broader punctum size range (0.5–0.7 mm) in a large cohort of patients with treatment-refractory, moderate-to-severe DED at a single tertiary center in Lebanon. We hypothesized that larger punctum sizes would be associated with greater tear film instability, as reflected by lower TBUT values, and that this relationship would be more pronounced in this uniformly refractory population. Additionally, this work contributes original clinical data from the Middle East—a region underrepresented in the DED literature.

## 2. Materials and Methods

### 2.1. Study Design and Ethical Considerations

This retrospective observational study was conducted in accordance with the principles of the Declaration of Helsinki. Ethical approval was obtained from the Institutional Review Board of the University of Balamand. Given the retrospective nature of the study and full de-identification of all patient data prior to analysis, the requirement for individual written informed consent was waived per institutional policy. This manuscript adheres to the Strengthening the Reporting of Observational Studies in Epidemiology (STROBE) guidelines for cross-sectional and cohort studies [16].

Medical records from Advanced Eye Care Center, Beirut, Lebanon—a tertiary-level ophthalmology hospital—were reviewed for the period January 2016 through December 2024.

### 2.2. Study Population

Eligible eyes were required to: (1) carry a documented diagnosis of moderate-to-severe DED meeting TFOS DEWS II Level 2 or greater criteria [7]; (2) have failed sequential topical therapy including artificial tear lubricants, loteprednol etabonate ophthalmic suspension (Lotemax^®^; Bausch + Lomb, Bridgewater, NJ, USA), and cyclosporine 0.05% ophthalmic emulsion (Restasis^®^; Allergan, Irvine, CA, USA) for a minimum of four weeks each; (3) have subsequently received a silicone punctal plug as part of clinical management; and (4) have a documented TBUT measurement recorded at the time of plug assessment.

Exclusion criteria were: absent or unrecorded TBUT data; punctal anatomical abnormalities (entropion, ectropion, punctal stenosis, or atresia); active ocular infection or inflammation contraindicating plug insertion; prior permanent punctal occlusion or lacrimal surgery; and concurrent systemic immunosuppressive therapy known to affect the ocular surface.

### 2.3. Lacrimal Punctal Diameter Estimation

**Figure 1:**
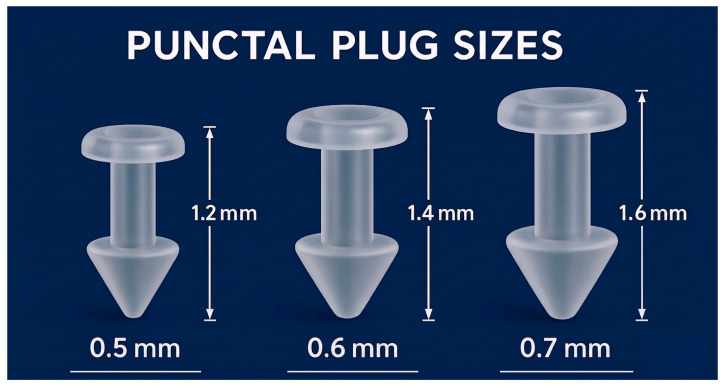
Schematic illustration of the three silicone punctal plug sizes used for lacrimal punctal diameter estimation in the present study. Plug shaft diameters of 0.5 mm, 0.6 mm, and 0.7 mm were trialed sequentially at the slit-lamp; the largest shaft diameter that could be inserted into the lower lacrimal punctum without forced dilation was recorded as the estimated lacrimal punctal diameter for that eye. The vertical dimensions annotated in the figure (1.2 mm, 1.4 mm, and 1.6 mm) represent the overall plug height, not the punctal diameter; readers should not interpret these measurements as reflecting punctal anatomy. Note: decimal separators in the figure labels use the standard decimal point notation is used (0.5/0.6/0.7 mm). Figure created for illustrative purposes only; plug dimensions shown are representative and not specific to any single manufacturer.

Lacrimal punctal diameter was estimated using a standardized plug-fitting procedure performed at the slit-lamp by a single senior ophthalmologist throughout the study period. Three silicone plug sizes were available: 0.5 mm, 0.6 mm, and 0.7 mm (Figure 1). The procedure involved sequentially trialing plug sizes and recording the largest diameter that could be fitted nonforcefully—without active punctal dilation—as the estimated punctal diameter for that eye. Plugs were placed exclusively in the lower lacrimal punctum of each eligible eye; upper puncta were not assessed or plugged. Each eye thus contributed a single lower-punctum diameter measurement to the dataset. The consistent use of a single examiner and a fixed plug assortment across the study period minimized procedural variability; however, formal assessment of intra-observer reproducibility was not performed, and this is acknowledged as a limitation.

### 2.4. Tear Film Assessment

Tear film stability was assessed by TBUT using the sodium fluorescein strip method. A fluorescein strip moistened with sterile saline was applied to the inferior conjunctival fornix; the patient was instructed to blink once and then keep the eye open while the examiner, using a cobalt blue slit-lamp filter, recorded the time in seconds from the last complete blink to the appearance of the first tear film break or dry spot. The TBUT measurement recorded at the encounter of punctal assessment was used for analysis. All measurements were obtained by the same clinical team using a consistent protocol across the study period.

### 2.5. Statistical Analysis

Continuous variables are reported as median with interquartile range (IQR) and mean with standard deviation (SD); categorical variables as frequency with percentage (%). Normality of TBUT distributions was assessed using the Shapiro–Wilk test. Given the non-normal distribution and the ordinal structure of the grouping variable, the Kruskal–Wallis H test was used as the primary inter-group comparison. Statistically significant results were followed by post hoc pairwise Mann–Whitney U tests with Bonferroni correction to control the family-wise error rate across three comparisons.

Spearman’s rank correlation coefficient (ρ) was calculated between lacrimal punctal diameter (treated as an ordered three-level categorical variable: 0.5, 0.6, 0.7 mm) and TBUT across all 312 individual eye-level observations, to quantify the strength and direction of the monotonic association. The significance threshold was set at α = 0.05 (two-tailed). Statistical analyses were performed using SPSS v.26 (IBM Corp., Armonk, NY, USA). The 312 eyes were derived from 156 patients, each contributing both eyes (bilateral design). The total patient count of 156 was documented at the time of chart review prior to de-identification; while patient-level linkage between paired eyes was not preserved in the de-identified analytical dataset, the bilateral nature of inclusion is known from the original clinical records. All reported statistical analyses (Kruskal–Wallis, Mann–Whitney U, and Spearman) were conducted at the eye level and do not account for within-patient inter-eye correlation. These *p*-values should therefore be interpreted with appropriate caution, as they may be inflated relative to analyses that formally account for clustering. Formal clustered analyses (e.g., generalized estimating equations or mixed-effects models with random intercepts per patient) could not be performed on the de-identified dataset; this constitutes a recognized and substantive methodological limitation, discussed further in Section 4.

## 3. Results

### 3.1. Study Cohort

A total of 312 eyes from 156 patients treated at Advanced Eye Care Center between 2016 and 2024 met all eligibility criteria and were included in the analysis. All included patients had both eyes assessed and plugged (bilateral inclusion); each eye contributed one lower-punctum diameter measurement. All eyes carried a diagnosis of moderate-to-severe DED at TFOS DEWS II Level 2 or greater, and all had failed sequential topical therapy with artificial tear lubricants, loteprednol etabonate, and cyclosporine 0.05% prior to punctal plug fitting. The cohort thus represents a uniformly treatment-refractory DED population in a real-world clinical setting. Cohort characteristics are described in the following paragraph.

The study cohort comprised 312 eyes from 156 patients (bilateral design; mean both eyes per patient). All 312 eyes satisfied the eligibility criteria by definition: each carried a diagnosis of moderate-to-severe DED at TFOS DEWS II Level 2 or greater and had failed sequential topical therapy with artificial tear lubricants, loteprednol etabonate, and cyclosporine 0.05% prior to lower punctal plug fitting. Eyes were distributed across lacrimal punctal diameter categories as follows: 0.5 mm (*n* = 15, 4.8%), 0.6 mm (*n* = 204, 65.4%), and 0.7 mm (*n* = 93, 29.8%). Detailed demographic and clinical variables (age, sex, DED subtype, systemic disease status, medication history, contact lens use, and prior ocular surgery) were not available in the fully de-identified retrospective dataset and could not be reported or adjusted for; this is acknowledged as a substantive study limitation.

### 3.2. Lacrimal Punctal Diameter Distribution and TBUT Values

The 312 eyes were categorized by estimated punctum diameter as follows: 0.5 mm in 15 eyes (4.8%), 0.6 mm in 204 eyes (65.4%), and 0.7 mm in 93 eyes (29.8%). All groups demonstrated clinically significant tear film instability consistent with moderate-to-severe DED.

**Figure 2:**
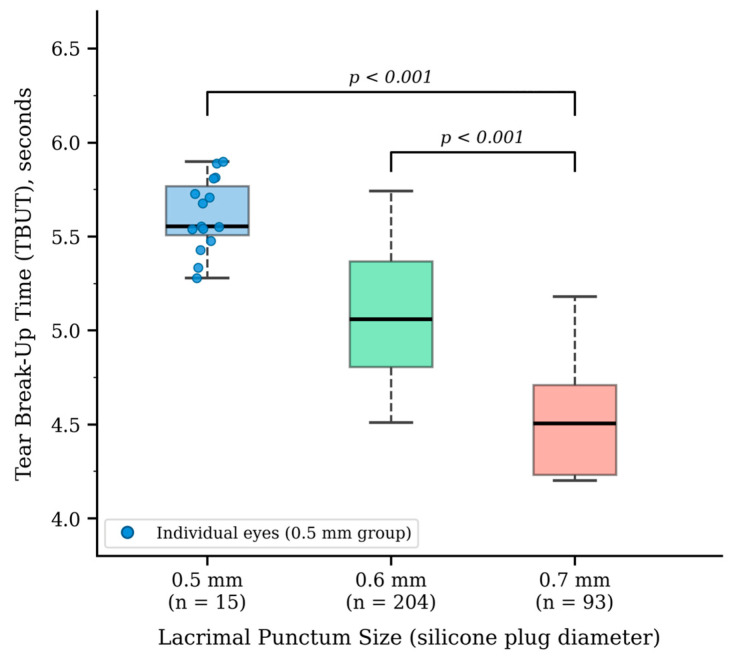
Boxplot illustrating the distribution of Tear Break-Up Time (TBUT, seconds) stratified by lacrimal punctum size category in 312 eyes with treatment-refractory dry eye disease. Horizontal lines within boxes represent group medians (0.5 mm: 5.55 s; 0.6 mm: 5.06 s; 0.7 mm: 4.51 s); box boundaries denote the 25th and 75th percentiles (IQR); whiskers extend to 1.5 × IQR; open circles represent outliers. Individual observations are shown for the 0.5 mm group (*n* = 15). Significance brackets with post hoc Bonferroni-corrected *p*-values are shown for pairwise comparisons. A progressive decline in TBUT with increasing punctum size is evident, consistent with the overall Kruskal–Wallis result (H = 140.1, *p* < 0.001) and Spearman rank correlation (ρ = −0.70, *p* < 0.000001). IQR, interquartile range; TBUT, tear break-up time.

A clear progressive inverse trend in TBUT was observed across increasing lacrimal punctal diameter categories. Median TBUT was 5.55 s [IQR 5.51–5.77] in the 0.5 mm group, 5.06 s [IQR 4.80–5.37] in the 0.6 mm group, and 4.51 s [IQR 4.23–4.71] in the 0.7 mm group. Although all groups fell within the range consistent with moderate-to-severe DED, those with larger puncta exhibited substantially lower TBUT values, indicating greater tear film instability. Detailed descriptive statistics are presented in Table 1, and the distribution is illustrated in Figure 2.

### 3.3. Statistical Association Between Lacrimal Punctal Diameter and TBUT

Kruskal–Wallis analysis confirmed a statistically significant difference in TBUT across the three lacrimal punctal diameter categories (H = 140.1, *p* < 0.001). Post hoc pairwise comparisons with Bonferroni correction demonstrated significant differences between all group pairs: 0.5 mm versus 0.6 mm (*p* < 0.001), 0.5 mm versus 0.7 mm (*p* < 0.001), and 0.6 mm versus 0.7 mm (*p* < 0.001), indicating that each increment in lacrimal punctal diameter category was associated with a statistically significant pairwise difference in TBUT. All analyses were conducted at the eye level and do not account for within-patient inter-eye correlation (bilateral design); the reported *p*-values should be interpreted with appropriate caution.

Spearman’s rank correlation, computed on all 312 individual eye-level observations with punctum size coded as an ordered variable, confirmed a strong, significant negative association (ρ = −0.70, *p* < 0.000001), indicating that larger lacrimal punctal diameter is robustly associated with greater tear film instability in this refractory cohort. The boxplot in Figure 2 illustrates the progressive decline in TBUT median and distribution across the three lacrimal punctal diameter categories.

## 4. Discussion

This retrospective study investigated the relationship between lacrimal punctum size and tear film stability in a cohort of 312 eyes with moderate-to-severe, pharmacologically refractory DED managed at a tertiary ophthalmology center in Lebanon. The central finding was a strong, statistically significant negative correlation between punctum size and TBUT (ρ = −0.70, *p* < 0.000001), corroborated by a Kruskal–Wallis test confirming highly significant inter-group differences (H = 140.1, *p* < 0.001) and significant pairwise separations between all three lacrimal punctal diameter categories after Bonferroni correction. The effect was clinically coherent: the 0.7 mm group had a median TBUT of 4.51 s, approximately one full second lower than the 0.5 mm group median of 5.55 s—a difference that is meaningful in the context of the TFOS DEWS II TBUT threshold of ≤10 s for DED and the narrow range within which moderate-to-severe cases typically fall.

These findings markedly extend the prior work of Chen et al. [15], who reported a modest correlation (ρ = 0.16) in a mixed-severity cohort restricted to plug sizes ≤0.5 mm. Two structural features of the present study explain the much stronger association: (1) inclusion was restricted to uniformly treatment-refractory patients, a population in whom the contribution of augmented tear drainage to DED pathophysiology is likely greatest; and (2) extending the plug size range to 0.7 mm captured a clinically important subgroup with substantially enlarged puncta not studied by Chen et al.

The mechanistic basis for the observed association is consistent with established lacrimal physiology. Tear drainage through the lacrimal puncta accounts for approximately 80% of total tear clearance under resting conditions [2,14,17,18]. Larger punctal diameters may facilitate increased drainage flow, shortening tear film residence time on the ocular surface and accelerating the development of tear film discontinuities—the functional substrate of TBUT reduction [9,10]. In the setting of ADDE, where basal lacrimal secretion is already insufficient, even modest increases in drainage capacity may disproportionately destabilize the tear film, consistent with the pronounced correlations observed in this refractory cohort.

These observations carry clinical implications worthy of cautious interpretation. The present data suggest that systematic documentation of lacrimal punctal diameter during plug-fitting in DED patients—a step that requires no additional equipment or time—may provide adjunctive clinical information. Patients with 0.7 mm puncta exhibited the lowest TBUT values and may warrant heightened attention to tear drainage as a contributing factor in their disease burden. However, the observed association between punctal diameter and TBUT may be confounded by variables that were not available in this dataset, including DED subtype (aqueous-deficient versus evaporative), age, sex, degree of ocular surface inflammation, meibomian gland dysfunction severity, Sjögren disease status, systemic medication exposure, and prior ocular surgery. Any of these factors may independently influence both punctal anatomy and tear film stability. The reported association should therefore not be interpreted as evidence of an independent causal relationship, nor should it be extrapolated to direct treatment decisions without validation in prospective, covariate-adjusted, multimodal studies. It is also important to distinguish statistical significance from clinical significance: the absolute difference in median TBUT between the 0.5 mm and 0.7 mm groups was approximately 1 s (5.55 s vs. 4.51 s), which, while statistically robust at the eye level, represents a modest absolute increment within the context of moderate-to-severe DED where all values fall below the TFOS DEWS II threshold of ≤10 s. Furthermore, as highlighted by Møller-Hansen et al. [14], surgical management of refractory DED is an evolving field encompassing amniotic membrane transplantation, salivary gland transposition, and emerging cell-based lacrimal gland therapies. Anatomical parameters such as punctal diameter, which can be recorded non-invasively during standard clinical encounters, may contribute to individualized stratification frameworks as this evidence base matures.

This study also demonstrates the potential value of punctum size assessment in resource-limited settings. Lebanon and the broader Middle East region are underrepresented in the DED literature [19,20]; the present findings from a Lebanese tertiary center contribute clinically applicable data relevant to this population and to other settings where advanced DED diagnostics (meibography, tear osmolarity measurement, MMP-9 testing) may not be universally accessible. TBUT, used here as the primary severity metric, is inexpensive, reproducible, and universally available, making the combined assessment of punctum size and TBUT a practical clinical tool adaptable to varied resource environments.

Several limitations merit acknowledgment. First, as a single-center retrospective analysis, the study is susceptible to selection bias inherent in a tertiary referral setting; the distribution of punctal diameters may not reflect that of the general DED population. Second, the bilateral design (both eyes from 156 patients included, contributing 312 eye-level observations) is a substantive methodological limitation. All analyses were conducted at the eye level without accounting for within-patient inter-eye correlation, which may inflate the reported *p*-values and overstate the precision of the estimates. The total of 156 patients was documented at chart review prior to de-identification; however, patient-level linkage between paired eyes was not preserved in the analytical dataset, precluding generalized estimating equation (GEE) or mixed-effects model analyses. Future studies should implement patient-level clustered analyses from the outset. Third, the small and markedly unbalanced 0.5 mm group (*n* = 15, versus 204 and 93 in the larger groups) limits the statistical stability of comparisons involving this category. Fourth, lacrimal punctal diameter estimation by sequential plug fitting is an indirect, observer-dependent, and discretized method (three levels only). All procedures were performed by a single senior ophthalmologist using a fixed plug assortment, which mitigates some examiner variability; however, formal intra-observer reproducibility was not assessed, and this constitutes a recognized limitation. Direct punctometry using anterior segment optical coherence tomography or calibrated digital slit-lamp imaging could provide more precise and reproducible measurements [21,22]. Fifth, TBUT served as the sole objective outcome. The multifactorial nature of DED is not fully captured by TBUT alone; future studies should incorporate multimodal assessments including OSDI questionnaire scores, Schirmer’s test, corneal fluorescein staining grade, tear osmolarity, and meibography, and should characterize DED subtype (aqueous-deficient versus evaporative) to allow subgroup analyses. Sixth, detailed demographic and clinical data (age, sex, DED subtype, ocular surface inflammation status, meibomian gland dysfunction severity, Sjögren disease status, medication history, contact lens use, and prior ocular surgery) were not available in the de-identified retrospective dataset and could not be used as covariates. Confounding by any of these variables cannot be excluded, and the observed association between punctal diameter and TBUT should not be interpreted as independent of these factors until confirmed in an adjusted analysis. Seventh, the “treatment-refractory” threshold of four weeks per agent is acknowledged as potentially insufficient to fully evaluate the cyclosporine response, which typically requires three to six months of continuous use for maximal immunomodulatory effect; patients documented as treatment failures at four weeks may have responded with prolonged therapy. This definition reflects chart documentation in a real-world clinical setting and should be interpreted accordingly. Finally, the cross-sectional design precludes causal inference; longitudinal prospective studies are required to determine whether punctum size independently predicts DED trajectory or modifies the response to punctal occlusion therapy.

## 5. Conclusions

In this retrospective, treatment-refractory DED cohort from a Lebanese tertiary center, larger lacrimal punctal diameter was significantly associated with lower TBUT values, as evidenced by a strong Spearman rank correlation (ρ = −0.70) and highly significant Kruskal–Wallis group separation (H = 140.1, *p* < 0.001) with all three pairwise comparisons remaining significant after Bonferroni correction. These findings suggest that lacrimal punctal diameter estimation during therapeutic plug fitting may offer a practical and cost-free adjunct to standard DED evaluation. However, the absolute TBUT differences between groups were modest, the study design was retrospective and bilateral, demographic covariates were unavailable, and TBUT was the sole severity metric. These findings should therefore be interpreted as hypothesis-generating rather than practice-defining. Prospective, multimodal, patient-level analyses—incorporating clustered statistical methods, comprehensive DED phenotyping, and adjustment for clinical and demographic confounders—are needed to establish whether lacrimal punctal diameter functions as an independent clinical indicator of DED severity and a reliable guide to individualized management decisions.

## Figures and Tables

**Table 1:** Tear break-up time (TBUT) values stratified by lacrimal punctum size category.

Lacrimal Punctal Diameter	*n* (%)	Median TBUT [IQR], s	Mean TBUT ± SD, s	TBUT Range, s	Post Hoc *p*-Value vs. 0.5 mm ^1^
0.5 mm	15 (4.8%)	5.55 [5.51–5.77]	5.61 ± 0.19	5.28–5.90	Reference
0.6 mm	204 (65.4%)	5.06 [4.80–5.37]	5.09 ± 0.34	4.51–5.74	<0.001
0.7 mm	93 (29.8%)	4.51 [4.23–4.71]	4.52 ± 0.29	4.20–5.18	<0.001

Overall: Kruskal–Wallis H = 140.1, *p* < 0.001; Spearman ρ = −0.70, *p* < 0.000001. ^1^ Post hoc Mann–Whitney U tests with Bonferroni correction (three comparisons); all three pairwise comparisons were statistically significant (0.5 mm vs. 0.6 mm: *p* < 0.001; 0.5 mm vs. 0.7 mm: *p* < 0.001; 0.6 mm vs. 0.7 mm: *p* < 0.001). TBUT: tear break-up time; IQR: interquartile range; SD: standard deviation.

## Data Availability

The de-identified dataset supporting the conclusions of this article is available from the corresponding author upon reasonable request, subject to institutional data governance requirements.

## Abbreviations

The following abbreviations are used in this manuscript:

ADDE	Aqueous-Deficient Dry Eye
DED	Dry Eye Disease
EDE	Evaporative Dry Eye
IQR	Interquartile Range
OSDI	Ocular Surface Disease Index
SD	Standard Deviation
STROBE	Strengthening the Reporting of Observational Studies in Epidemiology
TBUT	Tear Break-Up Time
TFOS DEWS II	Tear Film and Ocular Surface Society Dry Eye Workshop II
